# The effect of ionising radiation on the physical properties of 3D-printed polymer boluses

**DOI:** 10.1007/s00411-021-00892-z

**Published:** 2021-01-22

**Authors:** Karolina Jezierska, Anna Sękowska, Wojciech Podraza, Helena Gronwald, Magdalena Łukowiak

**Affiliations:** 1 Department of Medical Physics, Pomeranian Medical University in Szczecin, Ul. Ku Słońcu 12, 71-023 Szczecin, Poland; 2 Departament of Propaedeutics, Physicodiagnostics and Dental Physiotherapy, Pomeranian Medical University in Szczecin, al. Powstańców Wielkopolskich 72, 70-111 Szczecin, Poland; 3 Department of Medical Physics, West Pomeranian Oncology Center, ul. Strzałowska 22, 71-730 Szczecin, Poland

**Keywords:** 3D printing, Radiotherapy, Material hardness, Polymer bolus

## Abstract

In recent years, a method for designing radiotherapy boluses using 3D printing technology has been established in the West Pomeranian Oncology Centre in Szczecin, Poland. The aim of the present study was to investigate whether the ionising radiation used in radiotherapy affects the physical properties of the printing material. Particularly, the purpose of this study was to determine the effect of a 60 Gy X-ray radiation dose on the hardness and dimensions of 3D-printed boluses. Four cuboids were printed on a Zortrax M200 printer with acrylonitrile–butadiene–styrene (ABS) polymer. All printed samples were exposed to 60 Gy of X-ray radiation delivered by a medical accelerator. After irradiation, changes in the hardness (using Vickers test) and dimensions of the prints were measured. The therapeutic X-ray dose had a minimal effect on the dimensions of the printed samples, resulting in a maximum contraction of only 0.4%. Changes of the hardness were not statistically significant. In conclusion, regarding the radiotherapy planning process, the application of this therapeutic X-ray dose does not significantly influence the hardness and dimensions of ABS-printed boluses.

## Introduction

Boluses are tissue-equivalent materials with specific shape that can be applied directly to the patient’s skin. They are widely used in radiotherapy to alter the dose distribution of ionising radiation, especially during irradiation of superficial neoplastic lesions. Boluses provide additional absorption and scattering of ionising radiation, resulting in a more homogenous dose delivered to the area being treated (Hogstrom and Almond 2006; Łukowiak et al. 2017, 2016; Yan et al. 2018; Stone et al. 2003; Khan 2003).

In Poland, most boluses are created manually using paraffin wax to fit the patient’s surface. Other materials available in some centres are polymeric gels or ultrasound transmission gels. The present study concentrates on 3D-printed boluses filled with paraffin or water. In recent years, a method for the production of radiotherapy boluses using 3D printing technology has been introduced in the West Pomeranian Oncology Centre in Szczecin, and Acrylonitrile–butadiene–styrene terpolymer (ABS) has been selected as a bolus material. ABS-printed boluses were more precise compared with paraffin wax boluses that were manually created, and this helped to improve treatment planning (Łukowiak et al. 2017, 2016). The use of 3D printing allows for the production of customised phantoms with a shape designed to fit the patient’s therapeutic needs and anatomy. The 3D-printed boluses, when filled with water or paraffin wax, possess properties similar to those of irradiated biological tissues. Because of the method and the material used to produce these boluses, however, there is growing concern that irradiation can cause them to contract or change their mechanical properties.

ABS is a thermoplastic polymer commonly used in 3D printing, as it can be moulded after heat treatment. Moreover, this material slightly changes its size while solidifying (Hakimian and Sulong 2012; Rahimi et al. 2014; Sreedharan and Jeevanantham 2018). So far it remained an open question whether the ionising radiation used in radiotherapy affects the physical properties of such 3D-printed boluses. Consequently, the objective of the present study was to assess the suitability of ABS for bolus printing by measuring the changes in hardness and dimensions of 3D-printed ABS samples after exposure to 60 Gy X-rays. Changes in the hardness of a material can result in a greater tendency to deformations, and even small changes may negatively impact dose distribution in irradiated tissues.

## Material and methods

**Fig. 1:**
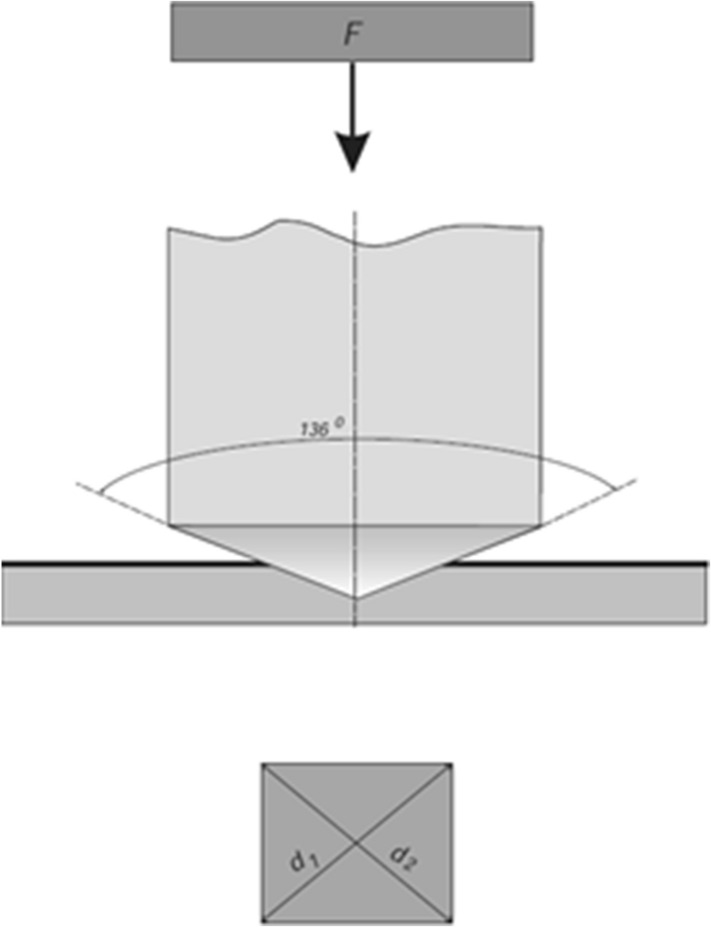
Schematic illustration of Vickers hardness test. *d* the average length of the diagonals d_1_ and d_2_ left by the indenter, *F* force applied to the indenter

Material hardness was evaluated using Vickers test, also referred to as the microhardness method. In this test, an indenter (diamond right square pyramid with a dihedral angle of 136°) is pressed onto the flat surface of the material with a specific force for a definite time (Fig. 1). The size of the resulting impression is measured with a microscope and used to determine the hardness of the material (Herrmann 2011; Chen et al. 2011; Shahdada et al. 2007; Franco et al. 2004; Asikuzun et al. 2012). The hardness number is determined by the ratio:

1 $$HV= \frac{F}{S}\left[\frac{N}{{mm}^{2}}\right]$$

where F is the applied force and S is the surface area of the resulting indentation, which can be determined by the formula:

2 $$S=\frac{{d}^{2}}{1.854}\left[{\mathrm{mm}}^{2}\right]$$

where d is the average length of the diagonal left by the indenter. Hence:

3 $$HV=0.189 \frac{F}{{d}^{2}}\left[\frac{\mathrm{N}}{{\mathrm{mm}}^{2}}\right]$$

The samples printed for the microhardness test were designed as hollow of cuboids using the Fusion 360 software. The file was exported to stereolithography format, which is compatible with the 3D printer’s software, and then printed with ABS polymer on a Zortrax M200 printer working on FDM technology. The parameters used to print were: nozzle temperature—260 °C, bed temperature—75 °C, layer thickness—0.14 mm, infill pattern—2 shells. Supports have not been applied.

Four identical samples were printed using this procedure and named I, II, R1 and R2. R1 and R2 were reference samples. The samples were stored at room temperature until further use.

Prints I and R1 were used for microhardness testing. Before testing, the surface of the samples was smoothed using sandpapers with 1200, 2400 and 4000 grit sizes. Using an unprepared print to measure microhardness is not accurate, as its surface is not smooth enough. The hardness of samples I and R1 was measured 30 times each, using a DURAMIN-40 AC3 hardness tester (Struers, Germany) with a load of 10 g applied for 10 s each time.

The dimensions (*x* and *y*) of samples II and R2 were measured 30 times each, using an electronic calliper with accuracy 0.02 mm and resolution 0.01 mm.

Prints I and II were irradiated with a single dose of 60 Gy, delivered with a Siemens ARTISTE 3 linear accelerator emitting X-rays at 6 MV. The measurements of microhardness and dimensions were repeated immediately after and 45 days after exposure, since the average duration of radiation treatment is 45 days for all prints.

The distribution of the data was assessed with the Shapiro–Wilk test. For normally distributed data, the statistical significance of the differences between means was evaluated using Student’s t-test. For non-normally distributed data, the Mann–Whitney *U* test was used (Statistica 13). Differences between the means were considered statistically significant at *p* < 0.05.

To check the dose distribution within the boluses, two randomly chosen treatment plans were analysed. Using the treatment planning system (TPS), boluses were planned, one with the volume of 51.2 cm^3^ and another one with the volume of 146.9 cm^3^. Then, a bolus shrink of 1 mm was simulated. The differences in dose distribution between the planed and simulated boluses were investigated. The mean, maximum and minimum dose for a planned Clinical Target Volume (CTV) were checked.

## Results

Tables 1, 2 and 3 present the statistics of the measured data. The changes in hardness (samples I and R1) were not statistically significant. The changes in dimensions for sample II were statistically significant only after 45 days (0.3% for x and 0.4% for *y* dimension). The measurements for reference sample R2 for dimension *x* were not statistically significant and for dimension *y*, the change was statistically significant only after 45 days after exposure, and was only 0.1% (0.03 mm). The *p* values for the comparison of the means are presented in Table 4. The results of the dose distribution comparison, for planned and simulated shrunken boluses are collected in Table 5.

**Table 1:** Vickers microhardness HV of samples Print I and reference sample Print R1, before irradiation and 1 and 45 days after irradiation with 60 Gy X-rays; *SD* standard deviation; *ND* normal distribution

	Microhardness $\left[\frac{\mathrm{N}}{{\mathrm{mm}}^{2}}\right]$ HV					
	Before/1/45 day		Δ1		Δ45	
	Print I	Print R1	Print I	Print R1	Print I	Print R1
Mean	13.2/12.9/12.8	12.8/12.8/12.6	0.3	0.0	0.4	0.2
Min	11.3/11.4/11.2	11.6/11.6/11.6	− 0.1	0.0	0.1	0.0
Max	14.8/14.4/14.5	14.3/14.1/13.7	0.4	0.2	0.3	0.6
SD	1.0/0.9/0.9	0.7/0.7/ 0.5	–	–	–	–
ND	Yes/yes/yes	Yes/yes/yes	–	–	–	–

**Table 2:** Changes in dimension x of samples Print II and reference sample Print R2, before irradiation and 1 and 45 days after irradiation with 60 Gy X-rays; *SD* standard deviation; *ND *normal distribution

	Dimension x					
	Before/1/45 days		Δ1		Δ45	
	Print II [mm]	Print R2 [mm]	Print II [mm]	Print R2 [mm]	Print II [mm]	Print R2 [mm]
Mean	30.0/30.0/29.9	29.9/29.9/29.9	0.0	0.0	0.1	0.0
Min	29.8/29.8/29.8	29.8/29.8/29.8	0.0	0.0	0.0	0.0
Max	30.1/30.1/30.0	29.9/29.9/29.9	0.0	0.0	0.1	0.0
SD	0.1/0.1/0.0	0.0/0.1/0.0	–	–	–	–
ND	Yes/yes/yes	Yes/yes/yes	–	–	–	–

**Table 3:** Changes in dimension y of samples Print II and reference sample Print R2, before irradiation and 1 and 45 days after irradiation with 60 Gy X-rays; *SD *standard deviation; *ND *normal distribution

	Dimension y					
	Before/1/45 days		Δ1		Δ45	
	Print II [mm]	Print R2 [mm]	Print II [mm]	Print R2 [mm]	Print II [mm]	Print R2 [mm]
Mean	25.2/25.2/25.1	25.1/25.1/25.1	0.0	0.0	0.1	0.0
Min	25.1/25.0/25.0	25.1/25.1/25.1	− 0.1	0.0	0.1	0.0
Max	25.3/25.6/25.2	25.1/25.1/25.2	− 0.3	0.0	0.1	− 0.1
SD	0.1/0.2/0.0	0.0/0.1/0.0	–	–	–	–
ND	Yes/no/yes	Yes/yes/yes	–	–	–	–

**Table 4:** Differences in microhardness HV, dimension *x* and dimension *y*, for samples Print I and Print II, and reference samples Print R1 and Print R2; Differences between the means were considered statistically significant at *p* < 0.05

Sample	Mean	*p* value			
		Dimension x	Dimension y	HV	
I	Before/1 day	–	–	0.17	
	Before/45 days	–	–	0.09	
II	Before/1 day	0.62	0.11	–	
	Before/45 days	< 0.05	< 0.05	–	
R1	Before/1 day	–	–	0.16	
	Before/45 days	–	–	0.06	
R2	Before/1 day	0.56	0.24	–	
	Before/45 days	0.76	< 0.05	–

**Table 5:** Comparison of dose distributions, for two planned and simulated shrunken boluses, for a shrinkage value of 1 mm. Δ*D*_mean_, Δ*D*_max_ and Δ*D*_min_ are the absolute values of difference in mean, maximum and minimum dose between planned and simulated boluses

	Bolus 1	Bolus 2
Δ*D*_mean_	0.3%	1.3%
Δ*D*_max_	0.4%	0%
Δ*D*_min_	1%	1.8%

## Discussion

This is a preliminary study, and further research will focus on performing a greater number of measurements using different irradiation parameters (type of radiation, dose, or beam energy). Based on the data obtained in the present study, it is suggested that even those changes in material hardness and dimensions that were statistically significant, are irrelevant to radiotherapy. Based on the results obtained, maximum shrinkage of the investigated boluses was 0.4% in all dimensions (Tables 2 and 3). The analysis of the planned TPS boluses shows that the maximum shrinkage, for a bolus with a length of 150 mm, should be 0.6 mm. However, a shrinkage of 1 mm, which is much greater than it should be, was simulated in the present study, because the TPS did not allow for the simulation of a smaller shrinkage. Even for a shrinkage of 1 mm, no significant differences in dose distribution were observed. For these changes to be relevant regarding treatment planning, the size of the print would have to be approximately 100 mm. Moreover, the changes in dimensions were observed 45 days after printing, not immediately after radiation exposure. Therefore, it could be that the observed reduction in size was not induced by radiation absorption, but represented just the natural contraction of the material. It can thus be suggested that only newly printed boluses should be used for treatment. However, more research is needed in this regard before a final conclusion can be drawn.

The ABS polymer is nontoxic and biologically inert with a density of 1.05 g/cm^3^ which is close to the density of soft tissue (1.05 g/cm^3^). Moreover, its atomic composition is similar to that of the human body. For these reasons, ABS seems to be an ideal material for bolus printing. The main disadvantages of using ABS are the fumes produced when printing and its non-biodegradability. Consequently, its use requires a proper ventilation system and generates a large amount of waste. Further work needs to be done to test other materials that could be used for printing boluses.

In conclusion, it is stated that ABS seems to be an appropriate material for creating 3D-printed boluses to be used in radiotherapy.

## Data Availability

Data will be available from authors upon request.

## References

[CR1] Asikuzun E, Ozturk O, Cetinkara HA, Yildirim G, Varilci A, Yılmazlar M, Terzioglu C (2012). Vickers hardness measurements and some physical properties of Pr2O3 doped Bi-2212 superconductors. J Mat Sci: Mat Electronics.

[CR2] Chen XQ, Niu H, Li D, Li Y (2011). Modeling hardness of polycrystalline materials and bulk metallic glasses. Intermetallics.

[CR3] Franco AR, Pintaúde G, Sinatora A, Pinedo CE, Tschiptschin AP (2004). The use of a vickers indenter in depth sensing indentation for measuring elastic modulus and vickers hardness. Mat Res.

[CR4] Hakimian E, Sulong AB (2012). Analysis of warpage and shrinkage properties of injection-molded micro gears polymer composites using numerical simulations assisted by the Taguchi method. Mater Des.

[CR5] Herrmann K (2011) Hardness measurement of plastic and elastomers. In: Hardness Testing: Principles and Applications. ASM International, pp 145–158

[CR6] Hogstrom KR, Almond PR (2006). Review of electron beam therapy physics. Phys Med Biol.

[CR7] Khan F (2003) Treatment verification. In: The physics of radiation therapy. 3rd ed. Phila-delphia: Lippincott Williams & Wilkins, pp 244–245

[CR8] Łukowiak M, Boehlke M, Lewocki M, Kot W, Matias D, Piątek- Hnat M, ElFray M, Jezierska K, Podraza W (2016). Use of a 3D printer to create a bolus for patients undergoing tele-radiotherapy. Int J Radiat Res.

[CR9] Łukowiak M, Jezierska K, Boehlke M, Więcko M, Łukowiak A, Podraza W, Lewocki M, Masojć B, Falco M (2017). Utilization of a 3D printer to fabricate boluses used for electron therapy of skin lesions of the eye canthi. J Appl Clin Med Phys.

[CR10] Rahimi M, Esfahanian M, Moradi M (2014). Effect of reprocessing on shrinkage and mechanical properties of ABS and investigating the proper blend of virgin and recycled ABS in injection molding. J Mater Process Technol.

[CR11] Shahdada SA, McCabea JF, Bullb S, Rusbya S, Wassella RW (2007). Hardness measured with traditional Vickers and Martens hardness methods. Dent Mater.

[CR12] Sreedharan J, Jeevanantham AK (2018). Analysis of shrinkages in abs injection molding parts for automobile applications. Mater Today-Proc 5(5). Part.

[CR13] Stone HB, Coleman N, Anscher MS, McBride WH (2003). Effects of radiations on normal tissue: consequences and mechanism. Lancet Oncol.

[CR14] Yan H, Guo F, Zhu D, Stryker S, Trumpore S, Roberts K, Higgins S, Nath R, Chen Z, Liu Wu (2018). On the use of bolus for pacemaker dose measurement and reduction in radiation therapy. J Appl Clin Med Phys.

